# P-1862. Rapid evolution of the *Staphylococcus aureus* Accessory Gene Regulator system under metabolic stress

**DOI:** 10.1093/ofid/ofae631.2023

**Published:** 2025-01-29

**Authors:** Edwin Chen, Leah Grady, Ian Weisner, Marla G Shaffer, Matthew J Culyba

**Affiliations:** University of Texas Houston, Houston , Texas; University of Pittsburgh, Pittsburgh, Pennsylvania; University of Pittsburgh, Pittsburgh, Pennsylvania; University of Pittsburgh, Pittsburgh, Pennsylvania; University of PIttsburgh, Pittsburgh, Pennsylvania

## Abstract

**Background:**

The Agr system is the master virulence regulator in *Staphylococcus aureus*. Active Agr signaling drives acute infections such as abscesses and endocarditis, while dysfunctional signaling is associated with persistent complications and antibiotic tolerance. Agr mutants have been isolated from nares and identified during persistent invasive infections. Thus, Agr loss-of-function mutants are considered an adaptive evolutionary strategy utilized by *S. aureus* to maintain its presence within hosts. The mechanisms underlying its selection, however, are not well understood. We recently identified genetic and environmental metabolic factors that selects for Agr loss.

Agr quorum sensing system in S. aureus

**Figure:**
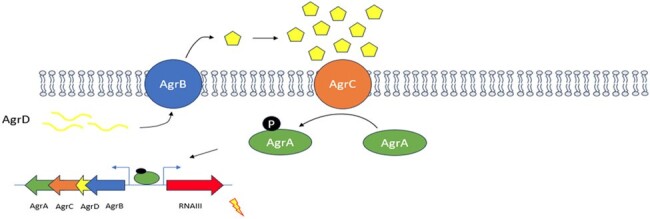

The Agr system is the master virulence regulator and is encoded by the agrBDCA operon. The auto-inducing peptide AgrD is matured and transported extracellularly by the membrane protein AgrB. At high enough population density, AgrD accumulates in concentration and is able to activate the surface receptor histidine kinase AgrC, which then signals intracellularly via phosphorylation of the response regulator AgrA. AgrA is a transcription factor that both feed-forwards the agrBDCA operon, as well as driving the expression of numerous virulence factors.

**Methods:**

Growth, *in vitro* evolution, and competitive fitness of USA300 JE2 MRSA strains were performed and analyzed in chemically defined media (CDM). The whole genomes of isolates that underwent *in vitro* evolution were sequenced using an Illumina platform. Agr hemolysis defect was assayed using sheep’s blood agar plates.

Growth assays of mutant strains in various CDM.

**Figure:**
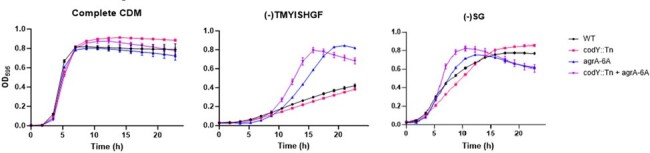

agr mutant strains have enhanced growth under specific amino acid stress.

**Results:**

We systematically analyzed the growth of a panel of USA300 JE2 MRSA strains (WT, *agr*, *codY*, *agr/codY*) in CDM lacking either single or multiple amino acids. We identified two specific CDM compositions that selectively enhanced the growth kinetics of strains containing *agr* mutations: (-)TMYISHGF and (-)SG. Growth competition assays in the same two CDM confirmed the fitness advantage of *agr* mutants. To next assess the physiologic relevance of *agr* mutations in these media conditions, we performed *in vitro* evolution and found that both media led to rapid accumulation of Agr loss. We further find that the *codY* strain, which is deficient in the global metabolic regulator CodY, also selects for *agr* loss independent of media conditions.

Competition assays of mutant strains in various CDM.

**Figure:**
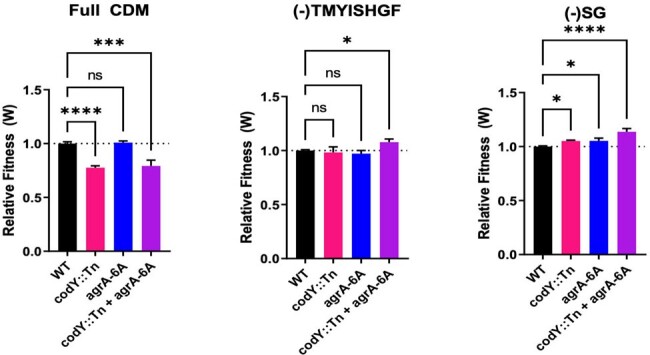

agr mutant strains have increased growth fitness under specific amino acid stress. Only agrA/codY had increased fitness compared to WT in CDM-TMYISHGF, whereas all mutant strains have increased fitness compared to WT in CDM-SG. Mean values were compared using one-way ANOVA (*, p<0.05; ***, p<0.001; ****, p<0.0001).

**Conclusion:**

Depending on its status, *S. aureus* Agr can either drive acute infections or establish a persistent presence. Our identification of genetic and environmental factors that facilitates this lifestyle switch provides valuable insight into how *S. aureus* adapts within the host during invasive disease. Elucidating the specific mechanisms involved will pave the way to the development of adjunctive anti-metabolism therapeutics that can provide protection against persistent infectious complications.

In vitro evolution of agr mutants in various CDM.

**Figure:**
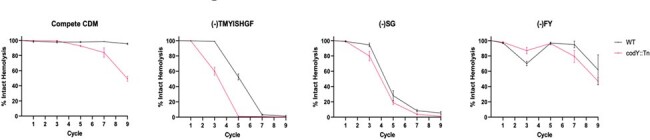

Tracking hemolysis loss – aka Agr loss – over time reveals both genetic and environmental influences on Agr loss. Genetically encoded codY mutations drive agr mutations via de-repression of QS signaling, whereas specific environmental nutrient stress also drives Agr loss.

**Disclosures:**

All Authors: No reported disclosures

